# Intraocular DHODH-inhibitor PP-001 suppresses relapsing experimental uveitis and cytokine production of human lymphocytes, but not of RPE cells

**DOI:** 10.1186/s12974-018-1088-6

**Published:** 2018-02-21

**Authors:** Maria Diedrichs-Möhring, Sandy Niesik, Claudia S. Priglinger, Stephan R. Thurau, Franz Obermayr, Stefan Sperl, Gerhild Wildner

**Affiliations:** 1 Section of Immunobiology, Department of Ophthalmology, University Hospital, LMU Munich, Mathildenstr. 8, 80336 Munich, Germany; 2 Division Virus-associated carcinogenesis (F170), German Cancer Research Center (DKFZ), 69120 Heidelberg, Germany; 3 Department of Ophthalmology, University Hospital, LMU Munich, Mathildenstr. 8, 80336 Munich, Germany; 4 Panoptes Pharma GmbH, Reisnerstr. 34/1, 1030 Vienna, Austria

**Keywords:** Experimental autoimmune uveitis, Rat model, DHODH-inhibitor, Lymphocytes, RPE cells, Cytokines, Chemokines, VEGF

## Abstract

**Background:**

Uveitis is a potentially blinding inflammatory disease of the inner eye with a high unmet need for new therapeutic interventions. Here, we wanted to investigate the suppressive effect of the intraocular application of the small molecule dihydroorotate dehydrogenase (DHODH)-inhibitor PP-001 on experimental relapsing rat uveitis and furthermore determine its effect on proliferation and cytokine secretion of human peripheral blood lymphocytes (PBL) and human retinal pigment epithelial (RPE) cells in vitro.

**Methods:**

Spontaneously relapsing uveitis was induced in rats by immunization with interphotoreceptor retinoid-binding protein (IRBP) peptide R14. PP-001 was injected intravitreally after resolution of the primary disease to investigate further relapses. Proliferation and metabolic activity of phytohemagglutinin (PHA)-stimulated human peripheral lymphocytes with and without PP-001 and cytokine secretion were determined by XTT assay and bioplex bead assay. The RPE cell line ARPE-19 as well as primary human RPE cells treated with PP-001 or anti-vascular endothelial growth factor (VEGF) antibody bevacizumab were also investigated for metabolic activity and cytokine/chemokine secretion.

**Results:**

Injection of PP-001 into rat eyes reduced the number of relapses by 70%, from 20 relapses (57% of the rats affected) in the control group to 6 relapses (33% of the rats) in the treatment group. In human PBL cultures, PP-001 reduced the proliferation in a dose-dependent manner. The secretion of several cytokines such as IL-17, IFN-γ, and VEGF was suppressed by PP-001, as previously observed with rat T cells in the experimental autoimmune uveitis (EAU) model. In contrast, human RPE cells were not affected by PP-001, while the anti-VEGF antibody bevacizumab severely impaired the secretion of various cytokines including VEGF.

**Conclusions:**

For the first time, intravitreal injection of PP-001 demonstrated an effective, but transient reduction of relapses in the rat EAU model. In vitro PP-001 suppressed proliferation and cytokine/chemokine secretion of human lymphocytes, while neither human RPE cell line ARPE-19 nor primary RPE cells were affected.

## Background

Uveitis is a potentially blinding inflammatory disease of the inner eye. Autoimmune uveitis requires systemic immunosuppressive therapy of patients, which is frequently burdened with severe side effects. The choice of therapeutics for uveitis is modest, with corticosteroids, cyclosporine, and TNF-blocking agents as well as off-label use of methotrexate, azathioprine, and mycophenolates [1]; there is an urgent need for new and potent drugs causing less side effects. We have recently described the effect of oral administration of PP-001 (biphenyl-4-yl-carbamoylthiophene-2-carboxylic acid derivative), a new inhibitor of dihydroorotate dehydrogenase (DHODH), in two different experimental autoimmune uveitis (EAU) models in Lewis rats on inflammation as well as secondary neovascularization [2].

The two rat models of EAU are characterized by either a monophasic course of inflammation with neovascularizations as late sequel after immunization with S-Ag peptide PDSAg or a spontaneously relapsing-remitting course after immunization with IRBP peptide R14. In addition, we have shown the downregulation of proliferation and cytokine/chemokine secretion by autoreactive rat T cell lines. T cells specific for PDSAg secrete vascular endothelial growth factor (VEGF), and only PDSAg-induced EAU shows chorioretinal neovascularizations (CNV), despite a usually severe destruction of the retina in both types of uveitis and the fact that we observe CNV even in eyes with minor affection of the retina. We thus concluded that neovascularization in uveitis eyes is induced by the VEGF secretion of the intraocular T cells, since blocking T cells and their VEGF secretion by PP-001 prevented CNV in this type of EAU [2].

DHODH is an enzyme necessary for pyrimidine synthesis. PP-001 inhibits DHODH with a 150-fold higher potency than teriflunomide and especially targets activating T cells with an enhanced requirement for pyrimidines, which can only be covered by de novo synthesis [3]. Since systemic inhibition of T cell activation leads to a generalized immunosuppression, we are aiming at an intraocular application of PP-001 to only affect the local autoimmune response while sparing systemic immunity. Here, we show the effect of intravitreally injected PP-001 solution on the course of relapsing rat EAU. Moreover, we have investigated the in vitro effect of the small molecule on human peripheral lymphocytes and different retinal pigment epithelial (RPE) cells regarding proliferation and metabolic activity as well as cytokine and chemokine production. Since VEGF-production in PDSAg-specific, autoreactive rat T cells and chorioretinal neovascularizations in EAU can be suppressed by PP-001, we were interested whether PP-001 could also inhibit VEGF-secretion in human RPE cells [2]. For this purpose, we have compared the effect of PP-001 and the therapeutic VEGF inhibitor bevacizumab (Avastin®) on the human RPE cell line ARPE-19 and on primary human RPE cells, investigating cytokine and growth factor secretion. We looked at proliferation and metabolic activity and cytokine/chemokine secretion to compare it with the effect on activated human peripheral blood lymphocytes in vitro. While PP-001 efficiently downregulated proliferation and the secretion of a range of cytokines and chemokines in activated human peripheral blood lymphocytes, ARPE-19 cells and primary RPE cells were not affected. In contrast, the therapeutic VEGF inhibitor bevacizumab (Avastin®) dramatically suppressed the secretion of several cytokines by human RPE cells, including VEGF. Our data suggest that in uveitis patients PP-001 would specifically target intraocular T cells and their VEGF secretion and subsequent CNV induction, but without disturbing the homeostasis of basal VEGF-secretion of RPE cells that maintains the integrity of the choriocapillaris.

## Methods

### Induction of EAU in rats

Male and female Lewis rats (Lew/Orl Rj) were bred in our own colony or purchased from Janvier (Le-Genest-St-Isle, France). They were maintained under specific pathogen-free conditions with water and food ad libitum and used for experiments at the age of 6–8 weeks. Animals were immunized subcutaneously into both hind legs (50 μl/side) with a total volume of 100 μl emulsion containing 15 μg peptide R14 (human IRBP aa 1169-1191; Polypeptide Laboratories, France) and complete Freund’s adjuvant (CFA), fortified with mycobacterium tuberculosis strain H37RA (BD Biosciences, Germany) to a final concentration of 2.5 mg/ml. The time course of uveitis was determined by daily examination of animals with an ophthalmoscope and graded as described [4]. Clinical grading of the anterior chamber in brief, 0.5: enlargement of iris vessels, 1: peripupillar infiltration of leukocytes, 2: pupil covered with a fibrin clot, 3: anterior chamber hypopyon, and 4: anterior chamber hemorrhage. A relapse of EAU was defined as a score of > 0.5 after the resolution of the first course of inflammation and/or a period with a score of ≤0.5 or complete absence of all clinical signs of inflammation.

### Intraocular injection of rat eyes

Lewis rats (*n* = 30; age 6–8 weeks) were anesthetized using 0.5 mg/kg medetomidine s.c. Additional topical anesthesia with 0.4% oxybuprocaine eye drops was applied. After intraocular injection, sedation was antagonized with an s.c. injection of 2.5 mg/kg atipamezol. A stock solution of 35.5 mM PP-001 (MW 479.33; Panoptes Pharma GmbH, Austria, corresponding to 17 mg/ml) in absolute ethanol was diluted with sterile 0.9% saline to the final concentration of 0.5 or 0.8 μg/μl. Six microliters of solution with or without PP-001 (6.3/10.4 μM as indicated) were injected into the vitreous of both eyes with a 30G needle, controlled under an operation microscope. The perpendicular injection is penetrating the cornea without touching the lens or the retina. After injection, an antibiotic ointment was applied to the eyes to avoid infections.

### Intravitreal injection of rabbit eyes and measurement of intraocular PP-001 levels

Male Dutch belted rabbits of 13 to 18 weeks from Western Oregon Rabbit Company were used, and the experiments performed by Absorption Systems Inc. (ASI, San Diego, USA). They were housed in individual cages with water and food ad libitum. Procedures involving the care or use of animals in this study were reviewed and approved by the company’s Institutional Animal Care and Use Committee (IACUC). Animals were anesthetized with an i.m. injection of ketamine hydrochloride (30 mg/kg) and xylazine (5 mg/kg) for the injection procedure. Topical ocular anesthetics were used. The eyes and surrounding tissues were disinfected with 2% betadine ophthalmic solution and then rinsed with saline. Using a 30G needle, 25 μl PP-001 per eye was injected transsclerally 5 to 7 mm from the limbus. Both eyes were enucleated from animals euthanized by an intravenous injection of a commercial barbiturate-based euthanasia solution (150 mg/kg). The procedure was performed in compliance with the 2013 American Veterinary Medical Association (AVMA) Guidelines on Euthanasia. The eyes were rinsed with phosphate-buffered saline, and the retina was immediately dissected from each eye, snap frozen, and stored at − 70 °C until analysis. After thawing and addition of PBS and DMSO (1:1), the samples were homogenized and sonicated. Retina samples were homogenized using a MagnaLyser (Roche). The analysis of samples is based on determination of the analytes using tandem mass spectrometry after high-performance liquid chromatography. Pharmacokinetic parameters were calculated from the time course of the tissue concentrations. Pharmacokinetic parameters were determined with Phoenix WinNonlin (v6.3) software using a non-compartmental model with sparse sampling.

### Human peripheral blood lymphocytes

Collection of peripheral blood lymphocytes (PBL) from anonymized human donors with informed consent was approved by the ethics committee of the University Hospital, LMU Munich. Lymphocytes from heparinized blood of four healthy male donors (aged 26–35) were separated by a ficoll gradient and set up as triplicate cultures in RPMI1640 without phenol red supplemented with 4 mM l-glutamine, 1 mM sodium pyruvate, MEM non-essential amino acids, and 50 U penicillin/50 mg streptomycin (all from SIGMA, Deisenhofen, Germany) with or without 1% phytohemagglutinin (PHA) (Difco BD, Heidelberg, Germany) at a density of 1 × 10^6^/ml (cytokine detection in supernatants) or 2 × 10^6^/ml (XTT assays). Heat-inactivated human serum pool was added to a concentration of 5%. PP-001 was added to the cultures in concentrations of 3 μM (1.44 μg), 10 μM (4.8 μg), and 30 μM (14.4 μg) for XTT assay and 3 and 30 μM for the cytokine/chemokine detection for 72 h. Parallel assays were set up for XTT assay and collection of supernatants. Controls included cultures without test substance but with the diluent of PP-001.

### Retinal pigment epithelial cell line

The immortalized retinal pigment epithelial cell line ARPE-19 (ATTC® CRL-2302™) was cultured in DMEM/Ham’s F12 medium with stable glutamine supplemented with 50 U penicillin/50 mg streptomycin and 10%/FCS (Biochrom, Berlin, Germany). Confluent cells from passage 24 were transferred to 96-well microplates and cultured for 4 days as confluent cell layer before the medium was exchanged and PP-001 or anti-VEGF antibody bevacizumab (Roche Pharma AG, Grenzach-Wyhlen, Germany) were added to triplicate cultures. Controls without test substance but with the diluent of PP-001 were included. Culture supernatants were collected as described for human PBL and tested for cytokine/chemokine.

### Primary retinal pigment epithelial cells

The isolation of RPE cells (RPE 80.1 and RPE 81.6) from human cadaver eyes (anonymized donors) for scientific purposes was approved by the ethics committee of the Land Oberoesterreich. Human postmortem donor eyes were obtained from the Eye Bank of the Department of Ophthalmology at the Linz General Hospital (Linz, Austria) and processed within 4–24 h after death to obtain RPE cells. Isolation of human RPE cells was performed as described previously [5]. Human RPE cells of passage 4 from two different donors were used for the experiments. Cells were maintained in DMEM/Ham’s F12 (Biochrom) supplemented with 10% FCS. For stimulation experiments, the cells were confluently seeded on 96-well plates (passage 4) 4 days before adding PP-001 or bevacizumab to triplicate cultures for 72 h. Controls included cultures without test substance but with the diluent of PP-001. Supernatants were collected and tested for cytokines as described above.

### XTT-assays

XTT assays to determine proliferation and metabolic activity of human PBL after 72 h of culture were performed according to the manufacturer’s instruction (R&D Systems, Wiesbaden, Germany). Optical density (OD) was measured at 490 nm after 5 h of assay time.

### Bioplex bead assays to detect cytokines/chemokines

To determine cytokine secretion and to detect early and late secreted cytokines, 25 μl of supernatant was collected daily (24, 48, and 72 h) from each well of each triplicate, pooled, and immediately frozen at − 80 °C. Supernatants of triplicates from all time points (9 samples) were pooled immediately before testing with a Bioplex Pro Human Cytokine and Chemokine Assay (BioRad, Munich, Germany) for 29 analytes with a flow cytometry-based Luminex reader. Fifty microliters of supernatants were used for the assay according to the manufacturer’s instruction and tested for IL-1β, IL-1RA, IL-2, IL-4, IL-6, IL-7, IL-8/CXCL8, IL-9, IL-10, IL12p70, IL-13, IL-15, IL-17A, Eotaxin/CCL11, basic FGF, G-CSF, GM-CSF, IFN-γ, IP-10, MCP-1/CCL2, MIP-1α/CCL3, MIP-1β/CCL4, PDGF, RANTES/CCL5, TNF-α, VEGF, MCP-3/CCL7, MIP-3α/CCL20, and SDF-1α+β/CXCL12. The final values obtained in the bioplex analysis are calculated from the median value of fluorescence of at least 50 measured beads per analyte and sample.

### Statistics

Statistics was performed with InStat software using nonparametric (Mann-Whitney) tests (Table 1). For multiple comparison, Kruskal-Wallis test (nonparametric ANOVA) was used. *P* values ≤ 0.05 were regarded as significant.

**Table 1:** Effect of intravitreal injection of PP-001 on R14-induced relapsing EAU in rats

	Vehicle	PP-001
Mean max. clin. uveitis score primary disease (score 0 – 4 ± SE)	2.6 ± 0.1	2.7 ± 0.1
Mean max. clin. uveitis score at day of injection (score 0 – 4 ± SE)	0.5 ± 0.1	0.4 ± 0.1
Total no. of relapses post injection until day 6	20	6
No. of eyes with multiple relapses	2	0
No. of eyes with relapses	18 out of 30 eyes (60%)*	6 out of 29 eyes (21%)*
Relapses/animals	11/15 (73%)*	5/15 (33%)*
Mean max. uveitis score/relapse (score 0 – 4 ± SE)	1.1 ± 0.1	1.2 ± 0.3
Mean duration of relapse (days ± SE)	1.45 ± 0.11	1.17 ± 0.17
Mean appearance of first relapse (days post injection ± SE)	3.4 ± 0.5	4.3 ± 0.9
Mean severity of uveitis post injection until day 6 (± SE)	2.22 ± 0.25*	1.34 ± 0.22*

**Fig. 1:**
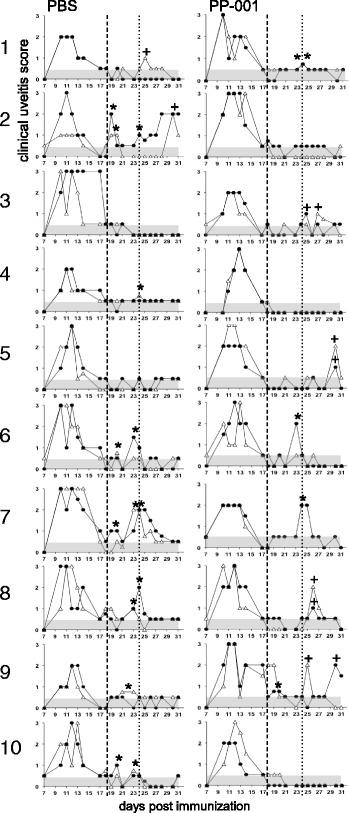
1–10 Clinical course of R14-induced relapsing EAU in rats after intravitreal injection of PP-001. Both rat eyes were injected once intravitreally with 6 μl PBS or 3 μg PP-001/6 μl PBS at day 18 (dashed line, resolution of primary inflammation) post immunization with R14-CFA. The clinical course is shown for each eye from each rat of both groups. The dotted line at day 24 defines the time period of therapeutic activity of intraocular PP-001. Relapses were defined as a clinical score of > 0.5 following a score ≦ 0.5. * Indicate relapses between days 19 and 24, **+** the relapses from day 25 until the end of the experiment. The gray areas show the negative/low inflammation scores that have to be exceeded for the definition of a relapse. Triangles: right eyes, black dots: left eyes. The right eye of rat PP-001/no. 7 was perforated during the injection and thus omitted from further evaluation. *N* = 10 rats per group; PBS *n* = 20 eyes, PP-001 *n* = 19 eyes after injection. Representative data are shown from experiment 1 of two independent experiments

“Mean severity of uveitis” is the mean of daily uveitis scores calculated over a certain period of time. One eye of the PP-001-treated group (rat 7/PP-001, see Fig. 1) was perforated during injection and not considered. Data are pooled from two independent experiments, Exp. 1, *n* = 10; Exp. 2, *n* = 5 rats/group**.** *p < 0.05

## Results

### Intraocular injection of PP-001

#### Clinical course of R14-induced relapsing EAU in rats after intravitreal injection of PP-001

**Fig. 2:**
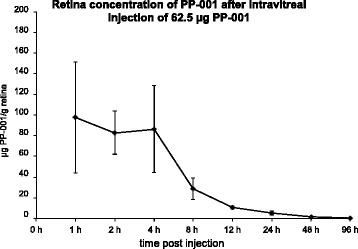
Pharmacokinetic profiles of PP-001 in rabbit eyes. Retinal concentration of PP-001 after intravitreal injection of 62.5 μg PP-001/rabbit eye (0 h). Tissues were collected at the indicated time points; PP-001 concentration is shown as nanogram per milligram/retina. *N* = 2 rabbits/4 eyes for each time point. Mean concentrations of PP-001 ± SD

PP-001 or PBS was injected intravitreally in two independent experiments with a final group size of 15 rats that had R14-induced relapsing EAU. The injection was performed when the primary clinical disease course had resolved to a score of ≦ 0.5 (day 18 post immunization), and disease progression was observed and graded until day 31 (Fig. 1). The clinical grading only considers signs of inflammation in the anterior part of the eye, since haze (score 1) and fibrin clotting the pupil (score 2) prevent investigation of the posterior segment. One eye of the PP-001-treated group (Fig. 1, no. 7) was perforated during the injection procedure and thus excluded from the study. Two independent experiments were performed with similar results; representative time courses from the first experiment are shown in Fig. 1, and the combined data from both experiments are given in Table 1. In the first experiment 3 μg PP-001 (6.3 μM) was injected into each eye of the treatment group (*n* = 10 rats, 20 eyes, Fig. 1). In the second experiment 5 μg PP-001 (10.4 μM) was used (5 rats, 10 eyes, time courses not shown). The rats of both treatment groups had a similar disease course prior to injection with maximal uveitis scores of 2.6 and 2.7, respectively, and a similar response to the treatment. In the PP-001-injected group, 29 eyes experienced 6 relapses, whereas in the vehicle-injected group, 20 relapses occurred in 30 eyes. Two eyes of the vehicle-injected group had 2 relapses, so 18 of 30 eyes in the control group were affected during the first 6 days post injection (days 19 to 24, Table 1). The reduction of relapses in the PP-001-treated group in relation to the control group was significant and substantial. In the control group, 73% (11/15 rats) of the animals had relapses, but only 33% (5/15 rats) in the PP-001-treated group. The number of eyes and the number of animals with relapses were significantly reduced in the PP-001-treated compared to the vehicle-treated group (*p* < 0.05), also the average daily intensity of inflammation from days 19 to 24 (*p* < 0.05) (PP-001, 1.34 ± 0.22; vehicle, 2.22 ± 0.25). The mean number of relapses/rat was 0.733 (CI 0.480–0.987) in the PBS-treated group and 0.33 (CI 0.063–0.604) in the PP-001-treated group; the mean number of relapses/eye was 0.633 (CI 0.365–0.902) in the control group and 0.241 (CI 0.076–0.407) in the PP-001 group. The average daily intensity of inflammation is shown as “severity of uveitis over time” and was calculated by the summation of the daily uveitis scores of all eyes in one group divided by the number of eyes and number of days (Table 1). PP-001 showed a significant transient suppressive effect on the relapses in R14-induced relapsing EAU. The duration of the effect is supported by a pharmacokinetic analysis of the retina after intravitreal injection (Fig. 2). It even suggests that a single local supply of efficacious dose levels of PP-001 for 24 to 48 h is sufficient to show an effect on relapses of EAU for 6 days.

#### Pharmacokinetic of PP-001 in rabbit eyes

**Fig. 3:**
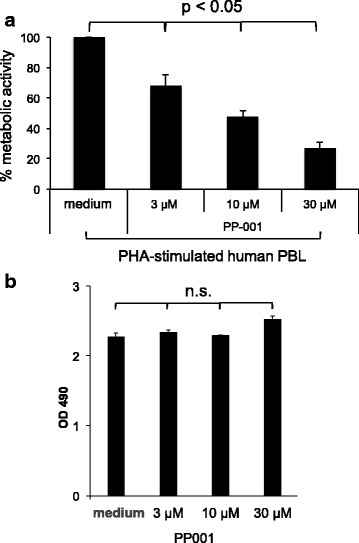
Proliferation and survival (metabolic activity) of PHA-stimulated human PBL and ARPE-19 cells treated with PP-001. **a** Inhibition of PHA-stimulated human peripheral blood cells from four different donors with PP-001, determined by XTT assay. OD 490 was measured after 72 h of PBL culture in triplicates with medium only, PHA and PHA with different concentrations of PP-001. Mean medium control values were subtracted from the mean values of stimulated cultures of each individual donor. Data of cultures stimulated with PHA only were defined as 100%; the percentage of activity (OD 490) is calculated in relation to the PHA control for each donor. Data show the means of % OD490 + SE from all donors. **b** XTT assay of triplicate cultures of ARPE-19 cells being confluent for 4 days and treated with either PP-001 or medium only. OD 490 was determined after 72 h of culture. Data are shown as mean OD 490 + SD

**Fig. 4:**
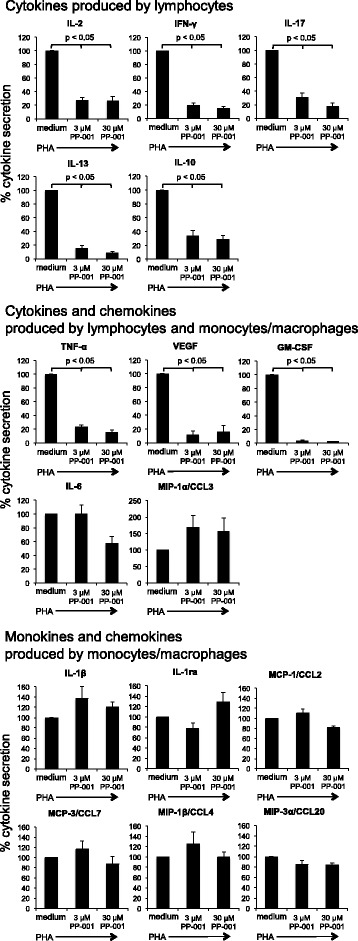
Cytokine secretion of PHA-stimulated human PBL treated with PP-001. Cytokine secretion of PHA-stimulated human PBL from four donors with or without PP-001 (3 and 30 μM, respectively). Supernatants from triplicate cultures were collected at three time points (24, 48, and 72 h after onset of cultures, in total nine samples) and pooled for the final bioplex analysis. Cytokine secretion of cultures with PHA only is defined as 100%; the percentage of secretion in cultures with PP-001 is respectively calculated in relation to the PHA-control for each donor. Data show the means of % secretion from four different donors + SE, *p* values are only shown for significantly different data

To further investigate the fate of PP-001 within the eye, pharmacokinetic studies of PP-001 in different ocular tissues were performed after intravitreal injection of 62.5 μg PP-001 into the eyes of Dutch belted rabbits. During the first 4 h post injection, the retinal concentration of PP-001 was stable between 83 and 98 μg/g retina and dropped thereafter (Fig. 2). A terminal half-life (*t*_1/2_) of 13.6 h was determined. After 48 h, the PP-001 concentration in the retina was still 3 μM, a concentration that was still highly suppressive for human lymphocytes, as shown in Figs. 3 and 4. PP-001 levels were comparable in choroid and in vitreous humor at all time points and AUC (area under the curve; which is integral in a plot of drug concentration in blood plasma vs. time) was 1/3 of that of the retina. After intravitreal injection of 62.5 μg PP-001, plasma concentrations were very low (Cmax = 41 ng/ml). Plasma levels were > 2000-fold lower than respective retina levels (maximal level 40.9 ng PP-001/ml), and potential systemic anti-inflammatory activity of PP-001 can be neglected at such low plasma concentrations. We have refrained from testing PP-001 levels in the inflamed eyes, since it has been published that there are no major differences expected in the pharmacokinetics between healthy and diseased eyes [6].

#### Effect of PP-001 on human lymphocytes and retinal pigment epithelial cells

We have previously shown that PP-001 efficiently reduced proliferation and metabolism as well as cytokine secretion of rat T cells in vitro, and one of the key cytokines affected by PP-001 was VEGF in PDSAg-specific T cells [2]. Thus, we investigated whether PP-001 would have similar effects on human lymphocytes and on retinal pigment epithelial cells.

#### XTT assay to determine metabolic activity

Metabolic activity and proliferation of human PHA-stimulated PBL were significantly suppressed in a dose-dependent manner by PP-001 as determined by XTT assay (Fig. 3a), while no effect was observed on human RPE cells ARPE-19 (Fig. 3b). Figure 3a shows the mean percentage of inhibition of PHA-stimulated PBL from four donors (the same as those assayed for cytokine secretion in Fig. 4) with increasing concentrations of PP-001.

#### Cytokine secretion

Cytokine secretion was measured in culture supernatants from PBL stimulated with PHA and treated with PP-001 in doses of 3 and 30 μM. Only data from those cytokines and chemokines with a level above the detection threshold of the assay from the four donors are shown in Fig. 4. Since the tested donor lymphocytes showed similar pattern in response to PP-001, but highly varied in the amounts of secreted factors, we have calculated the mean percentage of alteration of cytokine secretion compared to PHA stimulation only, the latter was defined as 100%. The highest amounts of secreted cytokines from PHA-stimulated PBL were determined for IFN-γ and TNF-α, followed by IL-6, IL-13, IL-2, and GM-CSF. The secretion of IL-8 from PBL is not shown, since it exceeded the detection level of the assay in all PHA-stimulated cultures. IL-17 secretion was moderate and even lower than IL-10, MIP-1a/CCL3, and VEGF. The lowest concentration of PP-001 (3 μM) already efficiently and significantly suppressed the secretion of the T cell-produced cytokines IL-2, IFN-γ, IL-17, IL-13, IL-10, TNF-α, VEGF, and GM-CSF, while the secretion of mainly monocyte-produced interleukins (monokines) IL-1β, IL-1ra, MCP-1/CCL2, MCP-3/CCL7, MIP-1β/CCL4, and MIP-3α/CCL20 was not suppressed by PP-001, suggesting that the T cell response, but not the innate immune response, was inhibited by treatment with PP-001. IL-6 and MIP-1α/CCL3 are produced by both T cells and myeloid cells and were not (MIP-1α/CCL3) or only slightly inhibited with 30 μM PP-001 (IL-6), suggesting that innate cells might have compensated for the suppressed T cell secretion of these factors (Fig. 4).

**Fig. 5:**
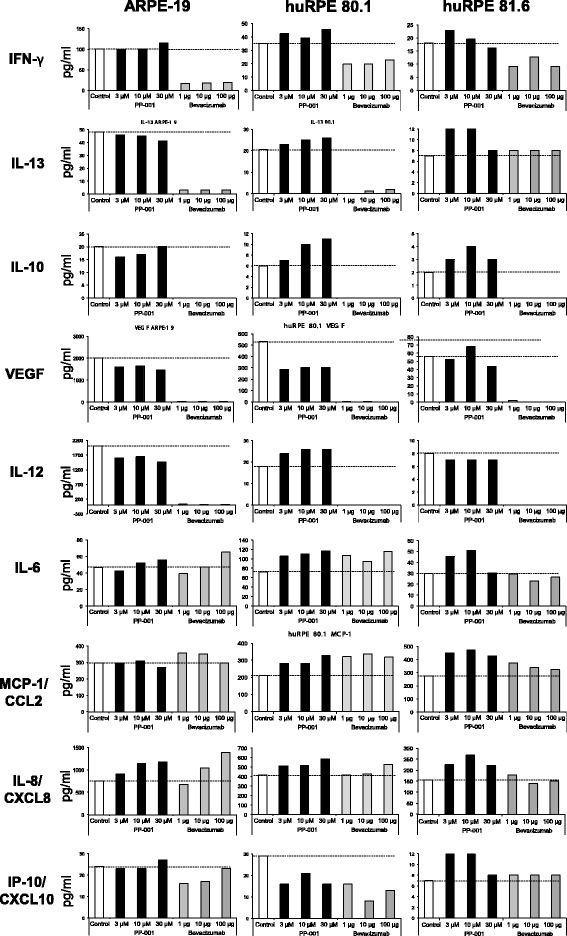
Cytokine/chemokine of human RPE cells treated with PP-001 or bevacizumab. Secretion of various factors by ARPE-19 cell line or two different primary human RPE cells (RPE 80.1 and RPE 81.6) cultured with different concentrations of PP-001 (black columns) or bevacizumab (μg/well). Supernatants from triplicate cultures collected at three time points (24, 48, and 72 h after onset of cultures, in total nine samples) were pooled for the final bioplex analysis. Data show the mean fluorescence of > 50 measured beads/analyte. “Control” (white columns) are the supernatants from cells cultured without inhibitor. Dashed lines indicate the baseline secretion of the cells according to the control culture

Human RPE cells were also co-cultured with various doses of PP-001 and bevacizumab as indicated (Fig. 5), and culture supernatant was collected daily to determine secreted cytokines, chemokines, and growth factors. As described for the human PBL above, only those cytokines/chemokines produced in amounts within the detection levels of the bioplex assay are shown in Fig. 5.

ARPE-19 cells and the primary human RPE cells secreted a number of cytokines, chemokines, and VEGF as the predominant growth factor. ARPE-19 cells had a higher baseline secretion of all factors compared to primary RPE cells from passage 4, except for IL-6, which was secreted in higher amounts by the primary RPE 80.1 cells (Fig. 5).

While PP-001 had almost no effect on the secretion of the tested factors and only slightly reduced the secretion of VEGF from ARPE-19 cells, bevacizumab efficiently suppressed IFN-γ, IL-10, IL-12, and IL-13 as well as VEGF secretion of ARPE-19 cells and the primary human RPE cells 80.1. IFN-γ, expressed at low levels in primary RPE, was only slightly diminished by bevacizumab, while the rather low IL-13 secretion was suppressed in RPE 80.1, but not RPE 81.6.

IL-8 production was enhanced in a dose-dependent manner with both, PP-001 and bevacizumab in ARPE-19, but not in primary RPE cells. IL-6, MCP-1/CCL2, and IP-10/CXCL10 secretion was not affected in any of the cells with both PP-001 and bevacizumab.

Despite the strong suppressive effect of PP-001 on lymphocytes, RPE cells remained unaffected. In contrast, bevacizumab, which is used for intraocular injections in case of uveitis neovascularizations, suppressed cytokine secretion of RPE cells. Bevacizumab thus might influence the function and role of RPE cells in the ocular immune privilege and their interaction with neighboring ocular structures like the choriocapillaris and choroid.

## Discussion

Autoimmune uveitis is usually treated with systemic immunosuppressive agents, especially when the posterior part of the eye is affected. To avoid side effects of general immunosuppression, local treatment by intraocular injection of anti-inflammatory or immunosuppressive drugs is desirable. Since we have shown previously that daily oral application of the small molecule DHODH-inhibitor PP-001 is highly effective in suppressing experimental autoimmune uveitis in two different rat models (relapsing-remitting and chronic disease with neovascularizations) [2], we decided to investigate its therapeutic effect after intraocular injection. We have chosen our relapsing-remitting model to prevent relapses with a single intravitreal application of PP-001 after the primary uveitis attack had resolved, and we could observe a strongly reduced relapse rate with significantly less intense inflammation in the PP-001-injected eyes within 6 days after treatment. This is the first time that the DHODH-inhibitor PP-001 was proven to be active not only systemically, but also after local, intravitreal injection.

The short-term effect was thought to be due to the limited retention time of the therapeutic substance in ocular tissues after intraocular injection, which was subsequently confirmed by pharmacokinetic studies of PP-001 in rabbit eyes. The duration of the therapeutic effect exceeded the actual presence of PP-001 most likely due to the suppression of local autoreactive T cells, which remain in the eyes even after the resolution of ocular inflammation and can initiate recurrences of inflammation [7, 8]. Tissue-resident T cells, which are reactivated later, or newly immigrating T cells would not be affected any more. This could explain the decreased effect on relapses of EAU later than 6 days post injection of PP-001.

After the first attack of uveitis had resolved to an activity score of 0.5 or less, some eyes showed increased inflammation at the time of injection of PP-001 in both groups. From the clinical point of view, this would be a typical scenario for the treatment in humans. Despite the increased activity, PP-001 still had a therapeutic effect. Overall, a single injection of aqueous formulated PP-001 was sufficient to achieve a local effect in the target tissue for several days. For long-lasting effects, the development of sustained release formulations is needed.

In rat models, PP-001 was shown to suppress in vitro proliferation of autoreactive T cells as well as secretion of certain inflammatory cytokines and chemokines [2]. Here, we demonstrate the suppression of proliferation of PHA-stimulated human PBL by increasing doses of PP-001, yet not below the level of the (unstimulated) medium control, which indicates that PP-001 was not cytotoxic. Cytotoxicity for PBL was also excluded by daily determination of the viability of cells, which was not diminished even at high concentrations (30 μM) of PP-001 (data not shown). The secretion of cytokines and chemokines of peripheral lymphocytes and RPE cells revealed that the levels of some factors secreted by PBL are strongly decreased with increasing doses of PP-001, while others remained unchanged or even increased. The latter is also arguing against general cytotoxicity of the small molecule. As observed with rat T cells [2], IFN-γ, IL-17, TNF-α, and VEGF secretion was also strongly suppressed by PP-001 in human PBL. Since these cytokines play pivotal roles in inflammation in uveitis the inhibition of intraocular T cells by an immunomodulating agent like PP-001 would be of high therapeutic relevance [8–10].

Also, IL-2, IL-10, GM-CSF, and even the Th2 cytokine IL-13 were strongly downregulated. It is concluded from our data that the DHODH-inhibitor PP-001 has more influence on the cytokine secretion of lymphocytes than on monocytes/macrophages. The reason is most likely found in the higher demand for de novo pyrimidine synthesis during the activation of lymphocytes, conveying the specificity of PP-001 for T and B cells [3].

VEGF can be secreted by activated T cells and induce Th1 responses [11, 12], and we have previously shown that only rats with PDSAg-induced EAU developed chorioretinal neovascularization (CNV). Retinal destruction is usually severe in both types of EAU (monophasic and relapsing), and we observe CNV even in those eyes with only minor destruction of the retina [2], so we hypothesize that CNV in uveitis is induced by the VEGF-secretion of the intraocular PDSAg-specific T cells rather than by ocular cells like RPE. This is supported by the fact that suppressing the T cells (and their VEGF secretion) by PP-001 was sufficient to prevent neovascularization in PDSAg-induced EAU [2].

The inhibition of VEGF secretion of human lymphocytes, but not of RPE cells by PP-001 might also be of therapeutic value for intraocular treatment in human uveitis cases with this sequel [13, 14]. In patients, neovascularization has been described in cases of choroiditis or chorioretinitis, where treatment with VEGF-inhibitors is very successful [15]. It is assumed that, similar to the generation of lesions in AMD, local VEGF-production induces CNV. In addition, retinal neovascularization may develop in retinal inflammation like vasculitis without angiographic signs of ischemia or alterations of the RPE or choroid [13]. It may be speculated that in these patients, increased VEGF levels originate from inflammatory T cells located in the vitreous, retina, or the perivascular space and induce sprouting of new vessels out of the retinal vasculature. This would be consistent with the neovascularization observed in rat EAU.

Since VEGF is a therapeutic target for wet age-related macular degeneration (AMD) in patients, we were interested whether PP-001 could also suppress VEGF secretion by human retinal pigment epithelial cells, which are the major source of VEGF in AMD.

The anti-VEGF antibody bevacizumab had a marked effect on the secretion of several cytokines and especially on VEGF-secretion of RPE cells, while PP-001 had only marginal effects. We speculate that the decreased secretion of many cytokines by RPE cells could result from the depletion of VEGF, an important autocrine survival factor of RPE cells, by the VEGF-neutralizing antibody [16]. In contrast, secretion of IL-8/CXCL8 was increased by both PP-001 and bevacizumab. Like VEGF, IL-8/CXCL8 also is an angiogenic [17] and anti-apoptotic survival factor for RPE cells [18], and both cytokines can induce neovascularization. VEGF even has the function of an autocrine survival factor for RPE cells [19, 20], and it is furthermore needed to maintain the vessels of the choriocapillaris [16, 21].

ARPE-19 cells express both VEGF receptors, VEGFR1 and VEGFR2, the latter is the major receptor to induce angiogenesis and to promote survival of RPE cells [22].

However, despite the positive effect of VEGF blockade on the leakiness of vessels and regression of neovascularization, severe damage like geographic atrophy and poor vision has been observed in AMD patients after extended treatment with anti-VEGF antibodies [23–25]. These adverse side effects would not be expected after PP-001 treatment, which is not affecting the RPE cells. Our data suggest that in patients with retinal vasculitis, PP-001 would specifically target intraocular T cells and their VEGF secretion and subsequent induction of neovascularization without disturbing the homeostasis of basal VEGF-secretion of RPE cells that is necessary to maintain the integrity of the choriocapillaris [21].

Regarding the effects of PP-001 on lymphocytes, our data suggest that the suppression of cytokine secretion is not a mere effect of suppression of proliferation and/or the metabolism of the T cells by impeding pyrimidine synthesis, but there must be additional mechanisms, probably due to posttranscriptional regulation, as described for cytokines and chemokines by Fan et al. [26]. Increased mRNA stability might explain the increase of IL-8 secretion under PP-001 treatment in ARPE-19 cells.

During treatment with PP-001 and bevacizumab, IL-8 might serve as an autocrine survival factor for RPE, as described for endothelial cells [18, 27]. Moreover, interaction of IL-8/CXCL8 with its receptor CXCR2, both expressed by RPE cells, upregulates VEGF mRNA and protein, which results in autocrine activation of VEGFR2 [28, 29]. Increased IL-8 production might therefore be a compensatory mechanism when VEGF secretion is inhibited.

## Conclusions

The small molecule DHODH-inhibitor PP-001 is a useful therapeutic agent for intraocular application in autoimmune uveitis. PP-001 could suppress lymphocytes without affecting ocular tissues like the retinal pigment epithelium. In the latter case, the integrity of the choriocapillaris and the choroid will be preserved, in contrast to the current treatment of intraocular neovascularization with VEGF blockers. Sustained release formulations for intravitreal application of PP-001 are currently developed to prolong the efficacy of the treatment to several months post injection.

## Abbreviations

CFA: Complete Freund’s adjuvant
CI: Confidence interval
CNV: Chorioretinal neovascularizations
DHODH: Dihydroorotate dehydrogenase
EAU: Experimental autoimmune uveitis
OD: Optical density
PBL: Peripheral blood lymphocytes
PHA: Phytohemagglutinin
RPE: Retinal pigment epithelium
VEGF: Vascular endothelial growth factor
